# Computational Modeling of Combination of Magnetic Hyperthermia and Temperature-Sensitive Liposome for Controlled Drug Release in Solid Tumor

**DOI:** 10.3390/pharmaceutics14010035

**Published:** 2021-12-24

**Authors:** Masoud H. H. Tehrani, M. Soltani, Farshad Moradi Kashkooli, Mohammadreza Mahmoudi, Kaamran Raahemifar

**Affiliations:** 1 Department of Mechanical Engineering, K. N. Toosi University of Technology, Tehran 19967-15433, Iran; masoud.tehrani@email.kntu.ac.ir (M.H.H.T.); farshad.moradi@email.kntu.ac.ir (F.M.K.); 2 Department of Electrical and Computer Engineering, University of Waterloo, Waterloo, ON N2L 3G1, Canada; 3 Centre for Biotechnology and Bioengineering (CBB), University of Waterloo, Waterloo, ON N2L 3G1, Canada; 4 Advanced Bioengineering Initiative Center, Multidisciplinary International Complex, K. N. Toosi University of Technology, Tehran 14176-14411, Iran; 5 School for Engineering of Matter, Transport & Energy, Arizona State University, Tempe, AZ 85287, USA; mahmoudi.m@asu.edu; 6 Data Science and Artificial Intelligence Program, College of Information Sciences and Technology (IST), Penn State University, State College, Pennsylvania, PA 16801, USA; kraahemi@gmail.com; 7 Department of Chemical Engineering, University of Waterloo, 200 University Avenue West, Waterloo, ON N2L 3G1, Canada; 8 School of Optometry and Vision Science, Faculty of Science, University of Waterloo, 200 University Avenue West, Waterloo, ON N2L 3G1, Canada

**Keywords:** targeted drug delivery, solid tumor, temperature-sensitive liposomes, magnetic nanoparticles, magnetic hyperthermia, multi-scale cancer modeling

## Abstract

Combination therapy, a treatment modality that combines two or more therapeutic methods, provides a novel pathway for cancer treatment, as it targets the region of interest (ROI) in a characteristically synergistic or additive manner. To date, liposomes are the only nano-drug delivery platforms that have been used in clinical trials. Here, we speculated that it could be promising to improve treatment efficacy and reduce side effects by intravenous administration of thermo-sensitive liposomes loaded with doxorubicin (TSL-Dox) during magnetic hyperthermia (MHT). A multi-scale computational model using the finite element method was developed to simulate both MHT and temperature-sensitive liposome (TSL) delivery to a solid tumor to obtain spatial drug concentration maps and temperature profiles. The results showed that the killing rate of MHT alone was about 15%, which increased to 50% using the suggested combination therapy. The results also revealed that this combination treatment increased the fraction of killed cells (FKCs) inside the tumor compared to conventional chemotherapy by 15% in addition to reducing side effects. Furthermore, the impacts of vessel wall pore size, the time interval between TSL delivery and MHT, and the initial dose of TSLs were also investigated. A considerable reduction in drug accumulation was observed in the tumor by decreasing the vessel wall pore size of the tumor. The results also revealed that the treatment procedure plays an essential role in the therapeutic potential of anti-cancer drugs. The results suggest that the administration of MHT can be beneficial in the TSL delivery system and that it can be employed as a guideline for upcoming preclinical studies.

## 1. Introduction

Nano-sized drug delivery systems (i.e., nanomedicine) have enabled efficient, sustained, and safer delivery of anticancer drugs through the encapsulation of drugs in nanoparticles. They can help to prolong drug half-life and reduce the exposure of the surrounding healthy tissue to the cytotoxic drug [1]. TSL combined with a hyperthermia technique offers a promising drug delivery system. The synergistic effects and feasibility of TSL-Dox in conjunction with mild local hyperthermia have been reported in the literature [2,3,4,5,6,7]. With this combination therapy, TSL is mostly intravenously injected and then enters the circulatory system to reach the ROI. TSLs were designed to release their contents in response to a threshold temperature of 40 °C and above [8]. Therefore, encapsulated drugs cannot be released in healthy tissues; additionally, the higher temperatures provided by hyperthermia trigger the release of the encapsulated contents at the tumor site. In addition to the acting role of triggering TSLs, the heat generated through hyperthermia can lead to the further death of cancer cells, especially in regions with low drug concentration. Consequently, a combination of drug treatment with heat can perform as a combinatorial thermo-chemotherapy system that is more beneficial than either of them separately.

Among several thermal therapy candidates, MHT, which uses magnetic nanoparticles (MNPs), exhibits acceptable results in clinical trials as a minimally invasive treatment [9]. MNPs, in particular iron oxides (Fe_3_O_4_), have shown great potential in biomedical applications due to their low toxicity and biocompatible nature [10]. A direct intratumoral injection of MNPs has shown reliable performance in preclinical and clinical studies due to providing a high concentration of particles [11,12,13]. The most significant advantages of MHT are deep tissue penetration, local and homogeneous heat, and the direct delivery of therapeutic heating to cancer cells [14]. However, MHT has received immense attention in recent years; adequate heating of the entire tumor volume is not possible due to many biological restrictions [15,16]. Thermosensitive liposomes have proven to be a successful tool in combination with local hyperthermia or thermal ablation because it can synergistically induce tumor elimination as a result of both high temperature and drug delivery [17,18]. The development of strategies that would allow MNP encapsulation within liposomes to induce local therapeutic hyperthermia has shown beneficial outcomes in previous studies [19,20,21,22]. The feasibility of the administration of TSLs combined with MHT has been reported in regard to the use of thermo-sensitive magnetic liposomes, which co-encapsulates MNPs and anticancer drugs [22,23]. Despite the recent advances in magnetoliposomes, some unsolved problems still exist. The insertion of nanoparticles into liposomes is limited by the thickness of the membrane, which is approximately 3.4 nm thick, so the incorporation of larger MNPs into liposomes is a formidable task [24,25]. Furthermore, the encapsulation of drugs, stability of nanoparticle-embedded liposomes, and purification of non-encapsulated magnetic nanoparticles are the other major problems [26,27]. There exist many computational and experimental studies on applying stand-alone MHT [28,29,30,31] or magnetoliposomes [32,33,34,35,36] to solid tumors, but there is a lack of studies on the intravenous administration of TSLs in combination with MHT.

The current study aims to develop a novel combinational therapy to overcome the mentioned limitations. With this motivation, TSL-Dox is intravenously administrated through bolus injection. MNPs are subsequently injected intratumorally to achieve localized heating under an alternative magnetic field (AMF). Due to the complex interplay between the drug delivery process and MHT and its impact on cancer cells, a multi-physical and multi-scale numerical model is developed to evaluate the performance of the suggested treatment approach. While different methods have previously been studied to improve the effectiveness of MHT, to the best of our knowledge, this is the first use of computational analysis in the study of MHT in the presence of TSL-Dox. In this regard, the integration and coupling of various mathematical equations, including bio-heat transfer, interstitial fluid flow, and drug transport are required. In the following, different aspects of effective parameters, including the initial dose, vessel wall pore size, the diffusion of MNPs, and the optimum time interval between the TSLs’ injection and MHT are examined in detail. The accuracy of the mathematical model is also evaluated against several previously published numerical and experimental studies.

## 2. Materials and Method

**Figure 1:**
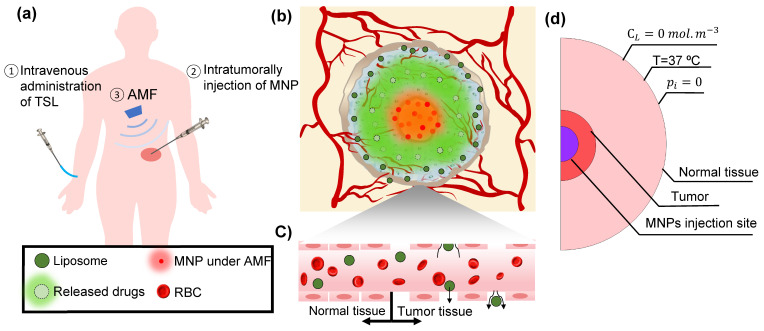
TSL-Dox delivery combined with MHT. (**a**) Schematic illustration of realizing the suggested combination therapy. The two nanoparticles have different localization: TSLs are injected intravenously and enter the tumor tissue through blood vessels. MNPs are injected intratumorally at the tumor core. MNPs increase tumor temperature when exposed to AMF. (**b**) The generated heat inside the tumor causes both cell death by heat and drug release from TSLs. Treatment outcomes of the proposed combination therapy depend on an acceptable concentration of released drug and temperature profile. (**c**) Due to the small vessel pore diameter in the healthy tissues, TSLs cannot pass through the vessel wall, and they mainly enter the tumor interstitial space. (**d**) The model geometry and boundary conditions.

This section summarizes the fundamental theory, assumptions, and mathematical methods used in the current study. The present study introduces a novel combination therapy to overcome the limitations of both conventional chemotherapy and MHT methods. Due to the complex interplay between hyperthermia and drug delivery systems, a computational model is developed to evaluate the performance of the suggested combinational therapy. Generally, the proposed model consists of three main steps, which are shown in Figure 1a. The first step is the administration of TSL-Dox through intravenous bolus injection. An optimum delay time between the first and the second step is needed to allow enough of the TSLs to concentrate in the tumor. The next step is the intra-tumoral injection of MNPs directly inside the tumor to reach the maximum concentration. The effect of MNPs’ diffusion on treatment outcomes is presented in Supplementary Method S1. The last step is the application of AMF, in which the heat generated by MNPs increases the temperature inside the tumor.

Figure 1b shows a schematic of the suggested combination treatment. MNPs localized at the tumor center increase the temperature when exposed to AMF. The generated heat can damage and kill cancer cells at the central part of the tumor. The temperature distribution around the injection site of MNPs is not high enough to kill cancer cells, but it may provide a threshold temperature (i.e., ~40 °C) for TSLs to release their cargo. The encapsulated drugs from TSLs can diffuse inside the tumor and kill the remaining cancer cells that had not been eliminated through MHT. TSL-Dox has less side effects over commonly used chemotherapies owing to their capacity for optional accumulation in tumorous tissue. Due to the enhanced permeability and retention effect, TSLs generally cannot enter healthy microenvironments (Figure 1c).

The tumor and its surrounding normal tissue are presented in Figure 1d. The radii of the tumor and the surrounding tissue are 1 cm and 3 cm, respectively. A total dose of 0.45 cm^3^ water-based ferrofluid with a 3.3% volume fraction is considered in this model, which creates a spherical injection site at the center of the tumor with a diameter of 0.5 cm [37]. As a general case, 10 nm MNP is used in this study, and an AMF with the frequency of 400 kHz and intensity of 13 kA/m is applied. In the following sections, different mathematical models employed in this study are described in detail.

### 2.1. Hyperthermia

Once MNPs are injected intratumorally, AMF is applied to increase the tumor temperature. Pennies bio-heat transfer equation is employed to describe the temperature field in the biological tissues during MHT as follows [38,39]:

(1) $$\rho C\frac{\partial T}{\partial t}=k\nabla^{2}T+\rho_{b}C_{b}\omega_{b}\left(T_{b}-T\right)+Q_{m}+\alpha Q_{MNP}$$

where ρ is the density, $C_{b}$ is the specific heat, *k* is the thermal conductivity, and *T* is the temperature. The mentioned parameters are listed in Table 1. The dissipated power by MNPs ($Q_{mnp}$) in porous media under an AMF is described by Rosenzweig’s theory. After the injection of MNPs, as the tissue is exposed to an AMF, heat is generated, and the therapeutic process starts. Heat generation by MNPs is determined by hysteresis loss and relaxation effects [40,41,42]. Magnetic hysteresis loss is negligible for small magnetic nanoparticles (generally smaller than 20 nm) because each nanoparticle becomes a single magnetic domain and shows superparamagnetic behavior with several important properties, such as negligible residual magnetism and coercivity [43]. The value of dissipated power by MNPs per unit volume is calculated as follows [40]:

(2) $$Q_{MNP}=\emptyset \mu_{0}\chi_{0}H_{m}^{2}f\frac{2\pi f\tau_{eff}}{1+{\left(2\pi f\tau_{eff}\right)}^{2}}$$

where $\mu_{0}=4\pi \times 10^{-7}\left(H/m\right)$ is the vacuum permeability; $H_{m}$ and ƒ are the amplitude and frequency of the magnetic field, respectively; and ∅ is the volume fraction of MNPs. Equilibrium susceptibility, $\chi_{0}$, strongly depends on the particle size [40]. The effective relaxation time, $\tau_{eff}$, in Equation (3) is a function of the Neel relaxation $\left(\tau_{N}\right)$ and Brown relaxation $\left(\tau_{B}\right)$ of the particles, which can be described as

(3) $$\tau_{eff}=\tau_{B}\tau_{N}/\left(\tau_{B}+\tau_{N}\right)$$

where $\tau_{B}=3\delta V_{H}/\left(k_{B}T_{s}\right)$, $\tau_{N}=\sqrt{\pi }\tau_{0}exp\left(\Gamma \right)/\left(2\sqrt{\Gamma }\right)$, $\Gamma =K_{eff}V_{H}/\left(k_{B}T_{s}\right)$, $K_{eff}$ is the anisotropy constant, $V_{H}$ is the volume of the coated nanoparticles, δ is the suspension viscosity, and $k_{B}$ is the Boltzmann coefficient [40].

We use the Arrhenius equation to model the cellular death in response to temperature elevation. We define a variable, DS, representing the FCKs by MHT alone.

(4) $$DS=exp-\left(\int_{0}^{t}Ae^{-\Delta E/RT\left(t\right)}dt\right)$$

*R* is the universal gas constant $\left(8.314\;J\cdot mol^{-1}\cdot K^{-1}\right)$, ΔE is the activation energy $\left(6.67\times 10^{5}\;J\cdot mol^{-1}\right)$, and A is the frequency factor $\left(1.98\times 10^{106}\;s^{-1}\right)$ [44,45].

### 2.2. Fluid Flow in Interstitium

Darcy’s law is used to calculate the velocity profile inside the interstitial space as [46,47]

(5) $$u_{i}=-k\nabla p_{i}$$

where $u_{i}$ and $p_{i}$ are interstitial fluid velocity (IFV) and interstitial fluid pressure (IFP), respectively. The tumor tissue is considered as a porous media with sinks and sources of mass due to fluid exchange between interstitial space and the lymphatic system. The continuity equation for an incompressible interstitial fluid within a porous medium considering source and sink is as follows [47]:

(6) $$\nabla \cdot v=\varphi_{B}-\varphi_{L}$$

where $\varphi_{B}\;\left(s^{-1}\right)$ and$\;\varphi_{L}\;\left(s^{-1}\right)$ represent the fluid flow rate from microvessels into the extracellular matrix and the fluid drainage rate by lymphatic vessels, respectively. $\varphi_{L}$ is assumed to be zero due to the absence of a lymph system inside the tumor [48,49]. $\phi_{B}$ is calculated through Starling’s law as [50,51]

(7) $$\varphi_{B}=\frac{L_{P}S}{V}\left(P_{b}-P_{i}-\sigma_{s}\left(\pi_{b}-\pi_{i}\right)\right)$$

The parameters used in these equations are the following: $L_{P}\left(\frac{cm}{mm\cdot \mathrm{Hg}\cdot s}\right)$, the hydraulic conductivity of the microvascular wall; $\frac{S}{V}\;\left(cm^{-1}\right)$, the vascular surface area per unit volume; $P_{b}\left(mm\cdot \mathrm{Hg}\right)$, vascular pressure; $\sigma_{S}$, the average osmotic reflection coefficient for plasma protein; $\pi_{B}\left(mm\mathrm{Hg}\right)$, the osmotic pressure of plasma; and $\pi_{i}\left(mm\mathrm{Hg}\right)$, the osmotic pressure of interstitial fluid. The interstitial transport properties used in the above-mentioned equations are defined and listed in Table 2.

### 2.3. Drug Transport

The spatiotemporal distribution of temperature resulting from the MHT is used as a stimulus to release the liposome’s cargo. Drug transport is defined by equations for encapsulated liposome drugs (l), free drugs (F), bound drugs (B), and the drugs internalized to cancer cells (I). The convection and diffusion mechanisms are accounted for to simulate the concentration of TSLs [54].

(8) $$\frac{\partial C_{l}}{\partial t}=D_{eff}\nabla^{2}C_{l}-\nabla \cdot \left(u_{i}C_{l}\right)-K_{EL}C_{l}+\Phi$$

in which $C_{l}$ denotes the concentration of TSLs, and $K_{EL}$ represents the release rate of the drug from TSLs, which depends on the composition of the liposome, the preparation method, and the temperature level. The relationship between the release rate and the given heating temperature is found to fit the first-order kinetics expression in existing experimental data [55]. If the temperature rises from 42 °C, the release rate can be considered as a constant value.

The last right-hand term in Equation (8), Φ, describes the drug extravasation through the microvascular network and also drug drainage via the lymphatic system. It is calculated as [46,53]

(9) $$\Phi =\Phi_{B}-\Phi_{L}$$

in which $\Phi_{B}$ is the drug supplied by the blood microvessels, and $\Phi_{L}$ is the drug drainage rate contributed by the lymph vessels. The drug-loss rate ($\Phi_{L}$) is neglected because there is no efficient lymphatic system in a tumor [6]. $\Phi_{B}$ is defined as follows [46,53,56]:

(10) $$\Phi_{B}=\varphi_{B}\left(1-\sigma_{f}\right)C_{pL}+\frac{PS}{V}\left(C_{pL}-C_{l}\right)\frac{Pe}{e^{Pe}-1}$$

in which *P* is the permeability of capillaries, $\sigma_{f}$ is the coefficient of filtration reflection, and $C_{pL}$ is the concentration of the drug in plasma. The ratio between convection and diffusion through the capillary wall is determined by the Peclet number ($Pe=\varphi_{B}\left(1-\sigma_{f}\right)/\frac{PS}{V}$).

The amount of free drugs in the interstitium can be calculated by Equation (11) as [57]

(11) $$\frac{\partial C_{F}}{\partial t}=K_{EL}C_{L}-\nabla \cdot \left(vC_{F}\right)+D_{f}\nabla^{2}C_{F}-\frac{1}{\varphi }K_{ON}C_{rec}C_{F}+K_{OFF}C_{B}$$

where $C_{F}$ is the concentration of free drugs in interstitial space, $D_{F}$ is the free drug diffusion coefficient, φ is the available volume fraction of tumor to drugs, and $C_{rec}$ is the concentrations of receptors on cell surfaces. $K_{ON}$ and $K_{OFF}$ represent constant rates describing drug binding and unbinding, respectively.

The concentration of the binding of Dox ligands to cell receptors in interstitium ($C_{B}$) is governed by [54]

(12) $$\frac{\partial C_{B}}{\partial t}=\frac{1}{\varphi }K_{ON}C_{rec}C_{F}-K_{OFF}C_{B}-K_{INT}C_{B}$$

where $K_{INT}$ is the rate of internalized drugs. The intracellular concentration ($C_{I}$) is a function of bound drug concentration as follows [54]:

(13) $$\frac{\partial C_{I}}{\partial t}=K_{INT}C_{B}$$

In the case of conventional chemotherapy, the equations for the concentrations of bound and internalized drugs are the same as those of TSL-Dox delivery, but the free concentration of the drug is determined as follows [54]:

(14) $$\frac{\partial C_{F}}{\partial t}=-\nabla \cdot \left(vC_{F}\right)+D_{f}\nabla^{2}C_{F}-\frac{1}{\varphi }K_{ON}C_{rec}C_{F}+K_{OFF}C_{B}+\Phi$$

An exponential curve fit, which is presented in Equation (15) over experimental data, is used to assess the performance of internalized Dox on overall cell survival rate [58]. Thus, the FKC is calculated as [59]

(15) $$FKCs=1-exp\left(-\omega \cdot C_{I}\right)$$

in which ω is cell survival constant.

Parameters for solute transport employed in drug delivery modeling are defined and listed in Table 3.

### 2.4. Boundary Conditions and Simulation Method

For the Darcy and mass transport equations, the continuity of pressure, velocity, and mass between the normal tissue and the tumor are imposed as the inner boundary conditions. Constant zero IFP is also applied to the exterior boundary of the tissue, as shown in Equation (21) [63]. Here, TSL-Dox is injected into the blood circulation system by bolus injection, so that the concentration of TSLs decreases as an exponential function [64].

(16) $$C_{pL}=Cp_{0}exp\left(-\frac{t}{k_{d}}\right)$$

where $k_{d}$ is the blood circulation decay, and $Cp_{0}$ is the initial dose of TSL-Dox, which is considered $0.5–1.5\;\mathrm{mol}/m^{3}$. All of the considered boundary conditions are listed in Equations (16)–(19):

(17) $${\left.-k\nabla p_{i}\right|}_{R_{n}^{-}}={\left.-k\nabla p_{i}\right|}_{R_{n}^{+}}$$

(18) $${\left.p_{i}\right|}_{R_{n}^{-}}={\left.p_{i}\right|}_{R_{n}^{+}}$$

(19) $${\left.\left(D_{eff}\nabla C+v_{i}C\right)\right|}_{R_{n}^{-}}={\left.\left(D_{eff}\nabla C+v_{i}C\right)\right|}_{R_{n}^{+}}$$

(20) $${\left.C\right|}_{R_{n}^{-}}={\left.C\right|}_{R_{n}^{+}}$$

(21) $${\left.p_{i}\right|}_{R}=0$$

In the intravenous injection, the drug or TSL concentration is assumed to be zero at the outer surface of the tumor. The temperature at the border of the normal tissue is considered to be 37 °C. The initial condition for IFP and the concentration are considered to be zero. The MHT process starts at a therapeutic body temperature of 37 °C.

In this study, the finite element method is used to analyze the coupled nonlinear set of governing equations via COMSOL^TM^ Multiphysics 5.5a software (COMSOL, Inc., Burlington, MA, USA). A time-dependent study is employed to solve this problem. The equations are solved over 72 h and consider 0.001 h time steps. Triangular mesh, with a total number of 32,747 elements, grew outward from the axis of symmetry with a minimum size of 1.2 × 10^−6^ m and a minimum element quality of 0.4238. All simulations are carried out on a computer equipped with an Intel Core i7 processor and 12 GB DDR3 RAM system.

### 2.5. Evaluation of Model Performance

**Figure 2:**
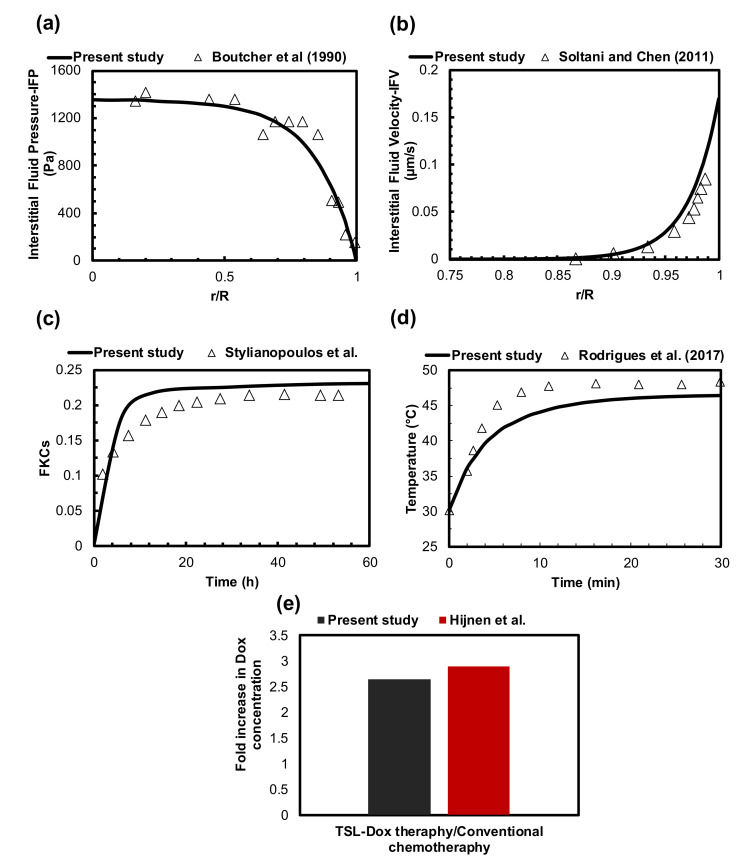
Validation of the current model performance. (**a**) The numerically calculated IFP distribution was validated with the experimental data from the literature. (**b**) The comparison of obtained IFV values with the theoretical amounts of previously published studies. (**c**) The value of FKCs compared to experimental results obtained 60 h after conventional chemotherapy taken from previously published studies. (**d**) Temperature comparisons between numerical simulation and experimental results of MHT on murine under the same conditions. (**e**) Qualitative verifications of TSL-Dox delivery in the present study and those in the literature.

The accuracy of our computational model is evaluated by comparing it with previous numerical and experimental studies. Since this problem consists of different equations, including the Darcy equation, bioheat transfer, and mass transfer, it is necessary to check the accuracy of each element. Comparing the computed distribution of IFP with the experimental data of Boucher et al. [65] in the exact same conditions showed a good consistency (Figure 2a). Additionally, an acceptable agreement is reported between obtained mean IFV values and the theoretical amounts presented by Soltani and Chen [66] based on the non-dimensional radial penetration (Figure 2b). The difference between results is 8% on average.

In this study, drug delivery equations were conducted similarly to the equations that were used by Stylianopoulos et al. [54]. They successfully validated their results by a comparison with in vivo data in murine mammary carcinomas. Thus, to examine the correctness of our simulation, the same parameter values of conventional chemotherapy were applied to the model. Figure 2c compares the value of FKCs over time. The results of this study have an acceptable agreement with those of the present study and those in the literature. The differences in tumor shape, tumor vasculature, inlet and outlet, normal tissue, etc., cause the differences between the results for the fraction of killed cells in our study and those reported by Stylianopolous et al. [37].

The accuracy of the bioheat transfer equation to calculate temperature distribution during MHT has been studied against an experimental investigation. Rodrigues et al. [67] examined MNP hyperthermia using a sarcoma 180 murine tumor, in which 3.9 mg of MNPs was injected intratumorally at three injection sites. The injection sites had depths of 5 mm. MHT was operated at the frequency of 301 kHz with a 220 G field amplitude in 30 min. The temperature profile at the surface of the tumor, resulting from the experimental study and the current simulation, is shown in Figure 2d. The temperature difference between the numerical and experimental results is 6% on average, which can be reduced to 4% when the temperature reaches a steady-state condition.

TSL-Dox delivery is of great importance in targeted drug delivery systems for tumor treatment. Since measuring concentrations in preclinical in vivo studies is very difficult, adequate experimental results are not available to validate the mathematical models. Therefore, we established qualitative verifications of TSL-Dox delivery via an in vivo study of Hijnen et al. [68]. Figure 2e compares the fold increase in DOX concentration between the present study and that in the literature. The fold increase in DOX concentration in our approach is 6% less than that in the approach of Hijnen et al. The difference in the fold increase in DOX concentration comes from the experimental approach versus the computational approach, which is marginal. It is also worth mentioning that the equations, parameters, and assumptions considered in this study have been confirmed in previous studies [69,70,71,72,73,74].

## 3. Results

### 3.1. Conventional Chemotherapy

**Figure 3:**
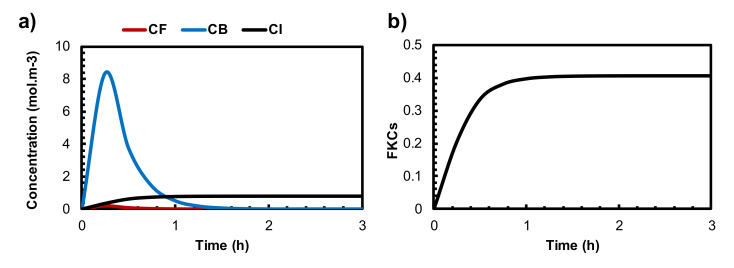
(**a**) The mean concentration of free, bound, and internalized drugs by the administration of conventional chemotherapy. (**b**) The FKCs during conventional chemotherapy.

The results of conventional chemotherapy can be used as a basis for evaluating the effectiveness of the combination therapy of TSL and MHT. Figure 3a shows the mean intratumoral concentration of free, bound, and internalized drugs over time. The concentrations of free and bound doxorubicin (Dox) follow the exponential decay function described in Equation (16). After 30 min post-injection, the average concentration of the bound Dox peak reaches 8.3 mol/m^3^ and then rapidly decreases due to the short lifetime of Dox in the body. The concentration of the internalized drug reaches its maximum value as soon as the treatment begins but remains constant. The maximum intracellular concentration of the drug reaches 0.78 mol/m^3^ after treatment, indicating that the tumor region is exposed to a low level of drug concentration. Consequently, just 40% of cancer cells are killed after treatment. This limited cancer cell disruption clearly indicates a low efficacy of chemotherapeutic drug delivery. Similar findings were reported in previous studies [75,76].

### 3.2. Treatment Efficacy of Localized MHT

**Figure 4:**
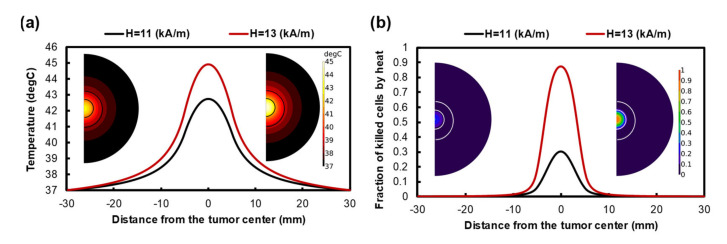
(**a**) Temperature profile after 60 min of heating with a frequency of 400 kHz and a magnitude magnetic field of 11 kA/m and 13 kA/m. Despite increasing the temperature level by using higher intensity, the temperature profile is limited to the injection site. (**b**) The fraction of killed cells after 60 min MHT.

Figure 4a shows a temperature profile generated by MHT with a frequency of 400 kHz and two different magnitudes of AMF amplitude. By applying 11 kA/m, the maximum temperature rises to 42.2 °C and 38.5 °C at the center of the injection site and tumor border, respectively. The temperature profile reaches its maximum value and remains constant 15 min after starting MHT. However, the FKCs resulting from the Arrhenius model indicate that the temperature is not high enough to kill cancer cells efficiently, especially at the tumor periphery. About 25% of cancer cells are eliminated at the tumor center and most of the tumor regions, and those surrounding the injection site remain alive after the treatment period.

Using a higher magnetic field amplitude can improve the heat generated by MNPs. Figure 4a,b indicate that increasing the magnetic field amplitude to 13 kA/m can improve the temperature level inside the tumor; therefore, 80% of cancer cells are killed at the tumor center. However, the temperature does not increase in the tumor regions adjacent to the injection site. Although using a high magnetic field eliminates most of the cancer cells at the injection site, the majority of the other parts of the tumor remain untreated. Increasing the magnetic field intensity did not cause the temperature gradient to expand in the tumor, so the effectiveness of the heat generated by MNPs was mainly restricted to their injection site in both high and low magnetic fields.

### 3.3. Quantifying the Anticancer Potential of Dox-Loaded TSLs Induced by MHT

**Figure 5:**
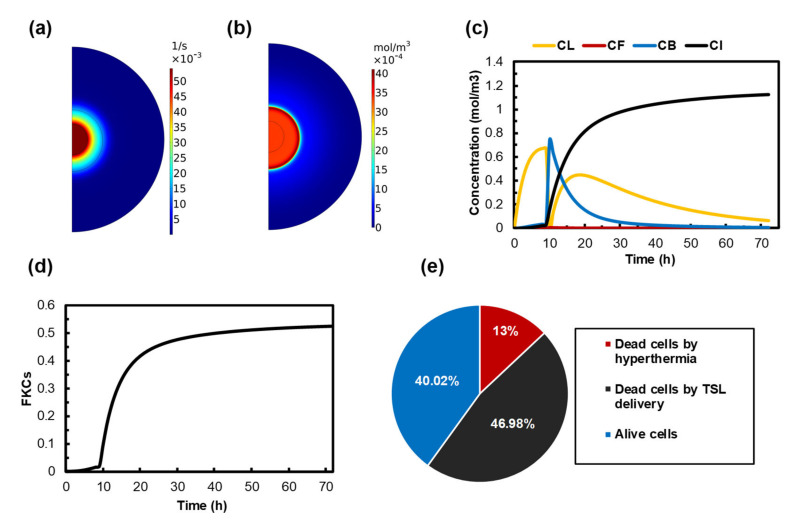
Treatment outcomes of MHT combined with TSL-Dox delivery by using 100 nm TSLs in a tumor with 200 nm vessel wall pore diameter. MHT is performed 9 h after TSL-Dox administration. (**a**) The release rate profile as a result of MHT. (**b**) Spatial distribution of free doxorubicin in the extracellular space of the tumor after MHT. (**c**) The concentration of TSL-Dox and free, bounded, and internalized Dox over time. The concentration of TSLs rapidly decreases after 9 h, because TSLs release the drug in response to temperature elevation. A dissimilar trend is shown for the concentration of bound and internalized drugs. (**d**) FKCs induced by release drug over time. (**e**) The effect of each part of the treatment on the final result.

In this section, the treatment outcomes of liposomal drug delivery with MHT are investigated for cancer therapy. The acceptability and feasibility of targeted drug delivery using MHT for hyperthermia-induced drug release from TSLs strongly depend on the amount of the drug that is released from TSLs during MHT. Figure 5a shows the spatial distribution of the release rate, which follows the temperature profile, with the highest value of 0.05 1/s achieved at the tumor center. This value is 0.035 1/s at the outer border of the MNPs’ injection site. The distribution of the drug release rate spreads to areas outside the injection site, covering twice the injection site radius size.

The therapeutic efficacy of TSLs strongly depends on their sufficient accumulation in the tumor. TSLs reach their maximum concentration 9 h after injection, so we performed AMF 9h after TSL delivery in our model (The effect of time interval between TSL delivery and MHT is investigated in Section 3.6). The extracellular unencapsulated drug concentrations in tumor and normal tissue, after 1 h of MHT in the presence of TSL-Dox, are presented in Figure 5b. The highest concentration is observed in the central region, where the tumor is directly heated by MNPs. The maximum concentration of free drug at the tumor periphery increases to ${4\;\times \;10}^{–3}{\;\mathrm{mol}/m}^{3}$. The result of free drug concentration indicates that the drug is released throughout the tumor.

In order to ensure that the amount of released drug within the tumor is sufficient, a long-term illustration of different drug concentrations is required. Figure 5c shows the distribution of $C_{L}$, $C_{F}$, $C_{B}$, and $C_{I}$ from the beginning of the treatment until 72 h post-treatment. With the bolus injection, the extracellular TSL concentration reaches its maximum 9 h after injection. Subsequently, it rapidly decreases due to the increase in temperature and the stimulation of TSLs to release their content. As expected, the variation in the bound Dox concentration follows the same pattern as that in the free Dox concentration, although the former is approximately 100-fold higher in magnitude. The concentration of bound Dox starts to increase and reach $0{.78\;\mathrm{mol}/m}^{3}$ at the end of the MHT, and it gradually reduces to zero as a result of a decrease in the intravascular concentration of TSL-Dox. The drug continuously enters the cellular space over time because of binding and internalization processes. The maximum intracellular concentration of Dox occurs when the bound drug concentration decreases to zero and reaches 1.2${\;\mathrm{mol}/m}^{3}$ at 72 h post-injection.

The ability of the drug to kill tumor cells plays an important role in selecting an efficient targeted drug delivery system. The time course of the survival fraction of cancer cells is presented in Figure 5d. The overall rate of killed cells is about 50%, indicating a much better drug delivery outcome compared to conventional chemotherapy.

Figure 5e shows the cellular response to the suggested combination treatment. Unsurprisingly, MHT alone has the least impact on the killing of cancer cells because the killing ability of MHT is restricted to the injection site. The outcomes of TSL-Dox delivery provide a great improvement in eradicating the tumor. About 50% of cancer cells are killed by the drug released from TSLs, which can, interestingly, enhance treatment efficacy. Ultimately, 40% of the tumor is affected by neither MHT nor TSL-Dox delivery. It is worth highlighting that 60% of the tumor is eradicated by a combination of these two methods.

### 3.4. The Effect of Vessel Wall Pore Size on Combination Therapy of MHT and TSL-Dox

**Figure 6:**
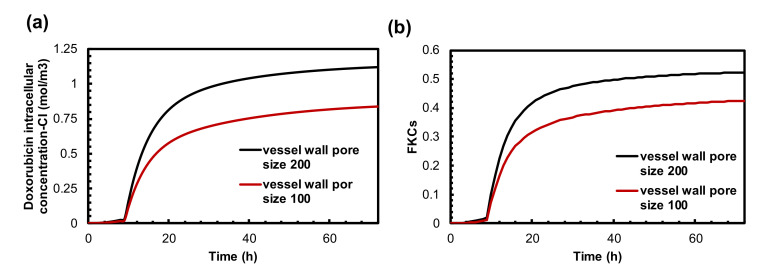
The effect of tumor vessel wall pore size on treatment efficacy of combination therapy. (**a**) Intracellular concentration of Dox is much higher in high permeable tumors. (**b**) FKCs over time. Therapeutic efficacy of TSLs in the presented combination therapy restricted in low permeable tumors. The efficacy of treatment is reduced as much as that of conventional chemotherapy.

The wall of microvessels is a barrier against efficient drug transport, which is dependent on the drug type and on the structural characteristics of the vessel pore sizes that may vary depending on tumor type and location [77,78]. Therefore, vessel wall pore size plays a crucial role in drug delivery, especially in nanomedicine. To study how changes in vascular pore size distribution can affect the presented combination therapy, we studied the treatment outcomes of two sizes of vessel pores (100 nm and 200 nm). Figure 6a demonstrates the intracellular chemotherapy concentration released from TSLs as a function of time. The highest concentration was achieved for hyper-permeable tumors (i.e., a 200 nm vessel wall pore size). For a 100 nm vessel wall pore diameter, drug uptake by cancer cells is reduced to $0.83\;\mathrm{mol}/m^{3}$. This value is 34% lower compared to a tumor with a 200 nm vessel wall pore size. As a result, the TSL-Dox delivery system induced by MHT can be more efficient for tumors with larger vessel wall pore sizes due to greater permeability.

The FKCs values resulting from TSL-Dox delivery are calculated for 200 nm and 100 nm vessel wall pore diameters as 0.52 and 0.42, respectively, indicating the importance of the effect of tumor microvascular characteristics on the final results.

### 3.5. Impact of TSL-Dox Dose

**Figure 7:**
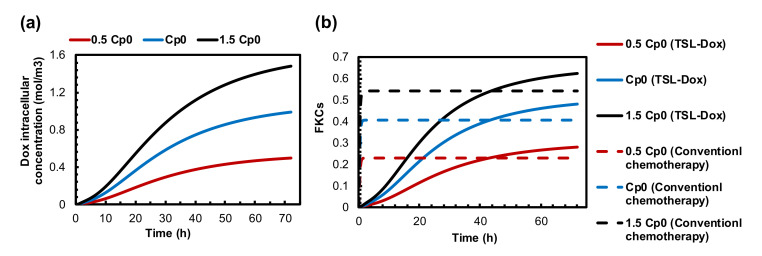
The effect of drug dosage on treatment efficacy of presented combination therapy. (**a**) Intracellular concentration. (**b**) FKCs over time.

Thus far, we could integrate two parameters of the suggested combination therapy. Another important parameter is the dose of TSL-Dox. Figure 7a,b shows the Dox internalized concentration and FKCs with three different doses of TSL-Dox. The concentrations of the internalized drugs and FKCs for a certain dose of TSL-Dox ($Cp_{0}$) are $1{.1\;\mathrm{mol}/m}^{3}$ and 0.53, respectively. With a 50% increase in injection dose, FKCs would be 0.63, increasing by 18%. The administration of a lower injected dose of TSL-Dox ($0.5\;Cp_{0}$) causes the concentration of the internalized drug and FKCs to reduce to $0.5\;\mathrm{mol}/m^{3}$ and 0.28, respectively. The effect of the initial dose on TSL delivery is the same as that of conventional chemotherapy. However, the efficacy of TSL delivery is higher with lower side effects. Increasing the injection dose from $Cp_{0}$ to $1.5\;Cp_{0}$ has shown the same improvement in both combination treatment and conventional chemotherapy. FKCs increase by 0.08, although the efficacy of TSL delivery is higher with lower damage to healthy tissues.

### 3.6. Optimization of the Time Interval between TSLs Administration and MHT

**Figure 8:**
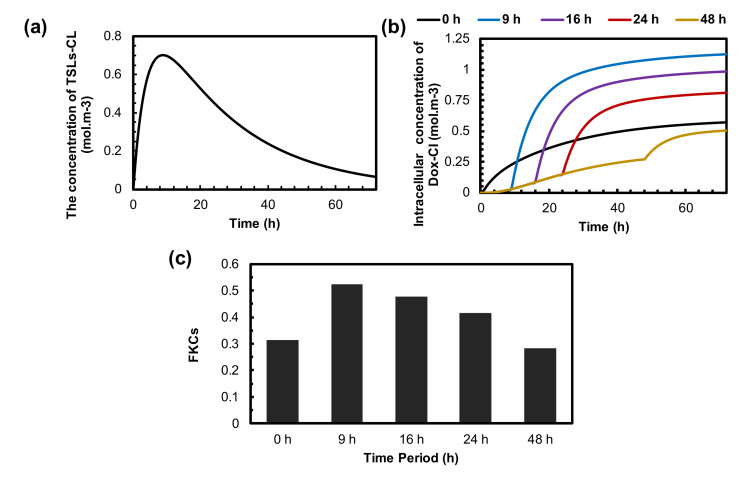
The effect of time interval between administration of TSLs and application of MHT on treatment efficacy of presented combination therapy. (**a**) The concertation of TSLs in tumor interstitial space. It reaches its maximum value 9 h after injection and then reduces to zero because of low half-life time in blood circulation. (**b**) The concentration of internalized drugs over time for different time intervals. The optimum time interval is achieved 9 h post-injection, in which TSLs efficiently accumulate in the tumor. (**c**) FKCs 72 h post-injection. The outcomes of combination therapy for different time intervals reveal that performing MHT at the right time can enhance treatment efficacy by up to 20%.

The treatment efficacy of TSL delivery strongly depends on the sufficient accumulation of TSL-Dox in the tumor when MHT is performed. This is because most drugs are released from TSLs during hyperthermia. The concentration of the TSLs that extravasate into the tumor through the microvessels is shown in Figure 8a. It shows that the concentration of TSL-Dox achieves the highest peak at 9 h post-injection and then gradually declines over time. This is because the concentration of TSLs in blood plasma reduces due to systemic clearance and transfer to other compartments (see Figure S2). The effect of the delay time between TSL-Dox administration and the application of MHT for five time intervals is demonstrated in Figure 8b,c. The maximum intracellular concentration of drugs is achieved when MHT is performed 9 h after TSL-Dox injection. The longer delay time (i.e., 24 h or 48 h) reduces the intracellular uptake of tumor cells due to the lower accumulation of TSL-Dox at the time of applying MHT.

The effect of delay time on TSL-Dox delivery is demonstrated in Figure 8c. Overall, FKCs reach 0.53 at a 9 h time interval, which is the best option in all investigated periods. This is because TSLs reach their peak concentration in the tumor before MHT. The higher delay time reduces the FKCs from 0.53 to 0.48, 0.41, and 0.21 for 16 h, 24 h, and 48 h deal times, respectively. The main reason is the reduction in the TSL-Dox concentration in the blood circulation and, consequently, the extracellular space of the tumor microenvironment. Moreover, we demonstrated that applying MHT directly after the injection of TSL-Dox reduces treatment efficacy by 20% because there is not enough time for the drug carriers to accumulate in the tumor.

## 4. Discussion and Conclusions

Previous studies have shown that both MHT and conventional chemotherapy have many limitations. Traditional anti-cancer drugs exhibit poor pharmacokinetics, limited bioavailability, and high toxicity, all of which restrict their clinical outcomes. In fact, tumor pathophysiology includes a highly dense extracellular matrix, which prevents efficient drug transportation in the interstitial space. The high level of the IFP at the tumor and the outward IFV at the tumor boundary reduce the drug penetration to the tumor. In addition to this, the absence of an efficient lymphatic system inside solid tumors is another essential factor that decreases drug delivery efficacy [71]. However, MHT generates a central zone of mild hyperthermic temperature (40–45 °C), surrounded by a non-destructive temperature [79]. The specific absorption rate (SAR) value also cannot significantly increase due to biological limitations [80]. Therefore, unheated cancer cells near the tumor border can increase the risk of tumor recurrence. TSLs, which are targeted drug delivery systems that release encapsulated drugs in response to temperature, have shown great potential to overcome the mentioned limitations. Combined with different localized hyperthermia methods, TSLs allow precise drug delivery with minimum side effects. The aim of the current study was to suggest a potential combination therapy to improve the shortcomings of both conventional chemotherapy and MHT. In this study, we proposed a multi-physics model to predict the performance of a new strategy of drug delivery based on an intravenous injection of TSL-Dox combined with regional hyperthermia made by the intratumoral injection of MNPs. The average concentration of the internalized drug and FKCs are considered as the main criteria for assessing the viability of the suggested approach.

Although chemotherapy is the key way to control cancer, it has been shown to have limited efficacy [81]. Among the different factors, insufficient drug penetration from the microvasculature and the side effects induced by chemotherapeutic drugs on healthy tissues and organs are major reasons behind the ineffectiveness of chemotherapy in patients [82]. The efficacy of chemotherapy depends on drug characteristics, such as binding affinity, cell-killing ability, and permeability. In the current study, Dox is considered for both chemotherapy and TSL delivery, with the characteristics that are listed in Table 3. As shown in Figure 3b, 40% of cancer cells are eliminated after conventional chemotherapy. As expected from previous studies, the treatment outcome is low due to the insufficient drug concentration inside the tumor.

MHT is a promising technique for targeted cancer therapy. Despite the recent progress in this field, its current clinical application is restricted due to several remaining challenges. In order to reach an effective temperature at the tumor, a relatively high nanoparticle concentration and high magnetic field strength and/or frequency are required. However, there is a significant risk arising from the toxicity effects introduced by MNPs, so a high concentration of particles is not possible. Moreover, the maximum frequency and amplitude of MHT are adjusted to ${H\;\times \;f\;<\;5\;\times \;10}^{9}{\;(A\;\times \;m}^{-1}{\;\times \;s}^{-1})$, which restricts the heat induced by MNPs [83]. Therefore, an adequate ablation of tumor margins is often impossible due to the vicinity of the normal tissues and the small heating zone. As depicted in Figure 4a, the effective heating zone that causes cell death is limited to injection margins; therefore, only about 13% of the tumor volume is affected by the hyperthermic temperature. The same conclusion has been reached in previous studies on the inadequacy of the heating zone due to thermal ablation or hyperthermia for large tumors [39,84,85]. An examination of the results of the two above-mentioned methods reveals that MHT is not able to eradicate cancer cells on the edge of the tumor; additionally, conventional Dox chemotherapy has been shown to have poor drug penetration. Ineffective drug penetration at the central regions of large tumors is more expected due to the existence of the necrotic core at the center [86].

Liposomes represent a versatile system for combination treatment strategies, which emerged as a potential solution to conventional chemotherapy problems because they can release their contents into the ROI [87]. Local hyperthermia has become the most widely used stimulus for the triggered release of liposomal drugs, providing a targeted control of drug release, which can enhance chemotherapeutic efficacy in many clinical settings [88]. In the current study, MHT is implemented as a stimulus for TSLs to release their cargo. It should be noted that the temperature in the vicinity of the injection site is not high enough to kill cancer cells, but it can increase the drug release rate from TSLs. As a result, the drug spreads to the area of the tumor that was not exposed to high temperatures. This result is obtained by comparing Figure 5a with Figure 5b. Figure 5b demonstrates that the drug spreads outside of the injection site, covering nearly an entire 1 cm tumor after MHT. Figure 5c indicates the final outcomes of the TSL-Dox delivery induced by MHT. A high intracellular concentration reveals the ability of MHT to provide enough temperature distribution for TSLs to release their contents. It is worth mentioning that released drugs are dispersed in the tumor region (due to diffusion and convection mechanisms in Equation (11)). In summary, the central part of the tumor, which comprises 13% of the tumor, is eliminated by hyperthermia. Furthermore, 47% of the remaining cells are eradicated by TSL-Dox delivery, and 40% of the cancer cells remain alive. A precise comparison between Figure 3b and Figure 5c indicates that the efficacy of TSL-Dox alone is 15% higher than that of conventional chemotherapy, which is due to the higher half-life of TSLs in the blood circulatory system, as well as the constant drug release rate from TSLs. MHT not only eliminates tumor cells via heat but also creates effective drug delivery through TSL-Dox.

An examination of the important parameters reveals features that can provide guidance toward an effective design of combination modality treatment. Among the different parameters, the permeability of the microvessels plays a key role in the performance of targeted drug delivery by TSLs. In this study, we developed a mathematical framework to study how changes in vascular pore size can affect the efficacy of the presented treatment method. The results indicate that TSL-Dox delivery in combination with MHT shows acceptable outcomes for high permeable tumors. As shown in Figure 6b in a tumor with a 100 nm vessel wall pore size, FKCs are reduced by 11% compared to a tumor with a 200 nm vessel wall pore size. In normal tissues, the size of the pores of the blood microvessels is less than 12 nm in diameter [89]. Therefore, TSLs with a size of 20 nm and larger do not extravasate to normal tissue, resulting in a drastic reduction in side effects when using TSLs as a nano-carrier. This is one of the major advantages of the suggested method over traditional treatment strategies.

The importance of the drug dose injected into a patient has been investigated for conventional chemotherapy in previous studies [90]. As shown in Figure 8, the changes in the dose of the injected TSL-Dox has a considerable impact on the treatment efficacy of the suggested combination method. With a 50% decrease in the injected dose, the FKCs are reduced by 21%. Due to the substantial effect of the injection dose, an appropriate dose of TSL-Dox must be selected based on the patient’s conditions.

Another important point shown in this study is the importance of the treatment procedure. The therapeutic effect of the localized drug delivery from TSLs in combination with MHT strongly depends on the adequate concentration of TSLs inside the tumor. The optimum period between TSL delivery and the application of MHT allows TSLs to accumulate in the tumor. The delivered amount of drug is directly related to the concentration of TSLs when MHT is applied. We found an optimum delay time of 9 h for 100 nm TSLs, which improve treatment efficacy by 20% compared to applying MHT just after the injection (Figure 8b). However, depending on the characteristics of the tumor and the TSLs, this value might change.

Due to some assumptions and simplifications considered in this study, the performance of each element is compared with previous studies. The error between the results of the current study and previously published experimental and numerical results is up to 8%. The influence of temperature rise on blood flow and permeability of the vasculature is not considered. Previous studies addressed the positive effect of hyperthermia on these parameters; thus, we can expect that the clinical results of the presented combination therapy will be better than those obtained in our simulation [91].

In conclusion, a multi-physics model was developed to predict the feasibility of combined TSL-Dox with MHT to increase treatment efficacy. The modeling framework described here indicates that the proposed approach could be exploited to deliver chemotherapeutic agents to the target site. The current study’s findings also revealed the importance of key parameters in the tumor microenvironment, such as tumor permeability, which is different for various tumor types. The results also highlight the importance of treatment schedules in determining treatment efficacy.

The model can be further developed by incorporating different injection strategies; these include an intravenous administration of TSLs or magnetic liposomes through continuous injection, direct injection of both MNPs and TSLs into the tumor, and intratumoral injection of chemotherapy drugs. The current model for microvascular transport can be extended to incorporate a more realistic vascular network as demonstrated by Kashkooli and Soltani [92]. In addition, specific cell killing models for different types of cancer cells and anticancer drugs could be employed to make the prediction more tumor specific.

## Figures and Tables

**Table 1:** Parameters of the biological materials.

Symbol	Quantity	Normal Tissue	Value [unit]		Reference
			Tumor	MNPs (Fe_3_O_4_)	
ρ	Density	1060	1040 (kg/m^3^)	5180 (kg/m^3^)	[40,41]
*k*	Thermal conductivity	0.59	0.57 (W/m°C)	528 (W/m°C)	[40,41]
*C*	Specific heat	3600	3600 (J/kg°C)	670 (J/kgK)	[40,41]

**Table 2:** Interstitial transport properties used in the modeling of the transport of MNPs in interstitium.

Symbol	Definition	Value [unit]	Reference
*L_P_*	Hydraulic conductivity of the microvascular wall	2.80 × 10^−7^ [cm/mmHg × s]	[52]
*K*	Hydraulic conductivity of the interstitium	4.13 × 10^−8^ [cm^2^/mmHg × s]	[52]
*S*/*V*	Surface area of blood vessels per unit tissue volume	200 [cm^−1^]	[52]
*P_B_*	Vascular fluid pressure	15.6 [mmHg]	[52]
π_B_	Plasma osmotic pressure	20 [mmHg]	[53]
π_i_	Osmotic pressure of interstitial fluid	15 [mmHg]	[53]
σ_s_	Average osmotic reflection coefficient for plasma proteins	0.82	[53]

**Table 3:** Parameters for solute transport employed in drug delivery modeling.

Symbol	Definition	Value [unit]	Reference
*D_F_*	Drug diffusion coefficient	$3.4\;\times \;10^{-6}\;\left[{\mathrm{cm}}^{2}/s\right]$	[60]
*P*	Microvessel permeability coefficient	$3\;\times \;10^{-4}\;\left[\mathrm{cm}/s\right]$	[61]
*K_ON_*	Constant of binding rate	$1.5\;\times \;10^{3}\;\left[M^{-1}\cdot s^{-1}\right]$	[54]
*K_OFF_*	Constant of unbinding rate	${8\;\times \;10}^{-3}\;\left[s^{-1}\right]$	[54]
*K_INT_*	Constant of cell uptake rate	${5\;\times \;10}^{-5}\;\left[s^{-1}\right]$	[54]
φ	Tumor volume fraction accessible to drugs	0.3	[54]
*C_rec_*	Concentration of cell surface receptors	${1\;\times \;10}^{-5}\;\left[M\right]$	[54]
ω	Cancer cell survival constant	$0.6603\;\left[m^{3}/\mathrm{mol}\right]$	[62]
*K_EL_*	TSL release rate at 42 ºC	$0.05409\;\left[s^{-1}\right]$	[55]

## Data Availability

The data presented in this study are available on request from the corresponding author.

## Supplementary Materials

The following are available online at https://www.mdpi.com/article/10.3390/pharmaceutics14010035/s1, Figure S1: Parameter study of three intervals between particle injection and applying AMF is performed to observe their impacts on treatment outcomes of MHT and TSL-Dox delivery, Figure S2: Dimensionless concentration of TSL-Dox in blood circulation system. The concentration gradually decreases from its initial value due to uptake of intravenously injected TSL-Dox by other compartments.
