# Supplementary material for: Computational Modeling of Combination of Magnetic Hyperthermia and Temperature-Sensitive Liposome for Controlled Drug Release in Solid Tumor
Source: Pharmaceutics. 2021 Dec 24;14(1):35. doi: 10.3390/pharmaceutics14010035 (PMC8778939; doi:10.3390/pharmaceutics14010035)
Supplement: Supplementary file 1 [file pharmaceutics-14-00035-s001.zip › pharmaceutics-1474425-supplementary.pdf]

pharmaceutics

Supplementary Materials: Computational Modeling of Combination of Magnetic Hyperthermia and Temperature-Sensitive Liposome for Controlled Drug Release in Solid Tumor

Masoud H. H. Tehrani, M. Soltani, Farshad Moradi Kashkooli, Mohammadreza Mahmoudi and Kaamran Raahemifar

Supplementary Method S1

Details on development of mathematical model describing the diffusion of MNPs before administration of AMF. The effect of MNP's diffusion on the treatment outcomes of MHT in combination with TSL-Dox delivery is investigated.

S1. Model extension to predict the diffusion of MNPs

The diffusion of the MNPs in the tumor is neglected in the main text. Because AMF is done immediately after the injection of MNPs to prevent their diffusion inside the tumor. As a result, as expected, the temperature increase remains very local. However, previous studies evidence particle diffusion after injection which can change temperature profile and therefore cell death rate as well as release rate [1, 2]. In this regard, we consider an additional step between intratumoral injection of MNPs and performing MHT. The concentration profile of MNPs inside the tumor after injection is determined by solving the convection-diffusion equation [3]:

$$\frac{\partial C_{MNP}}{\partial t} = D_{eff}\nabla^2 C_{MNP} - \nabla \cdot (u_i C_{MNP}) \quad \text{(S1)}$$

where D_{MNP} is the effective diffusion of particles with a diameter of d, in the tissue is calculated by the fiber matrix model as follows [4].

$$\frac{D_{eff}}{D_B} = exp\left[-\left(1 + \frac{d}{2a_f}\right)\eta^{1/2}\right] \quad \text{(S2)}$$

in which D_B denotes Brownian diffusion coefficient [5], $a_f = 200\ nm$ is the radius of the tumor matrix fibers, and $\eta = 0.66$ is the volume fraction of tissue fibers [6]. The volume fraction of the MNPs ψ, is given by the expression $\psi = C_{MNP}/\rho_{MNP}$ [7]. The last term on the right hand of Equation (1) represents the effect of change in volume fraction.

Figure S1 represents the dispersion of MNP's impact on both MHT and TSL delivery. The maximum temperature at the tumor center reduces from 45 °C to 42 °C, 40 °C, and 39 °C after 8 h, 24 h, and 48 h diffusion period, respectively. Cancerous cells have shown a considerable response to temperature reduction. The fraction of killed cancer cells reduces to 0.05 when MNPs disperse 8h before MHT. (Figure S1). As the temperature profile decrees below 42 °C because of the higher dispersion of particles, the temperature level is not high enough to damage cancer tissue efficiently. Arrhenius model predicts the percentage of dead cells nearly zero. The treatment outcomes of TSL-Dox delivery indicate that 8 h diffusion duration can have a minor positive effect on drug delivery, and the FKCs increase by 2%. Although, a longer diffusion period has shown an adverse effect on the release rate process. Because the value of FCKs reduces to 0.41, and 0.23 after 24 h, 48 h diffusion duration, respectively.

A better understanding of different aspects of the novel presented treatment is necessary to evaluate the potential of MHT to act synergistically with TSL delivery. In this study, a strong relationship was observed between diffusion of MNPs after injection and

treatment outcomes of both MHT and TSL-Dox delivery. Generally, diffusion of MNPs reduces the treatment outcomes of MHT. Slight correspondences are observed in the value of FKCs by chemotherapeutic drug between zero and short diffusion period. Because the TSLs considered in this study have a fast response to temperature. They release their content when the temperature exceeds 42 °C. short diffusion duration (i.e., 8 h) expands the temperature profile inside the tumor by holding maximum temperature up to 42 °C which is high enough to stimulus TSLs to release their content. Longer diffusion duration has shown a negative effect on treatment outcomes of TSL delivery as the release rate reduces significantly. It should be noted that this result depends on the characteristics of TSL, MNP, and tumor.

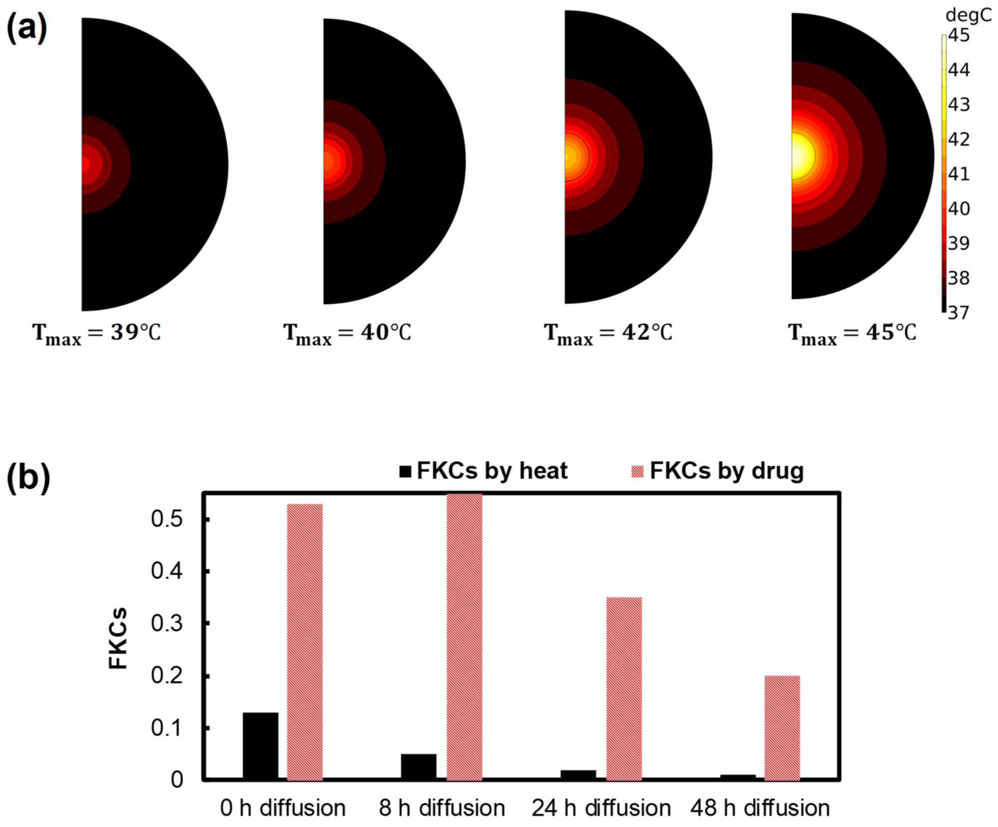

Figure S1. Parameter study of three intervals between particle injection and applying AMF is performed to observe their impacts on treatment outcomes of MHT and TSL-Dox delivery. (**a**) Temperature profile inside the tumor for three different intervals between particle injection and applying AMF. Both temperature level and the area affected by heat reduce due to lower accumulation of MNPs at injection site (**b**) The effect of diffusion duration on FKCs. Diffusion of MNPs more than 8 h have an adverse effect on both MHT, and TSL delivery. The results also show a minor improvement in TSL-Dox delivery.

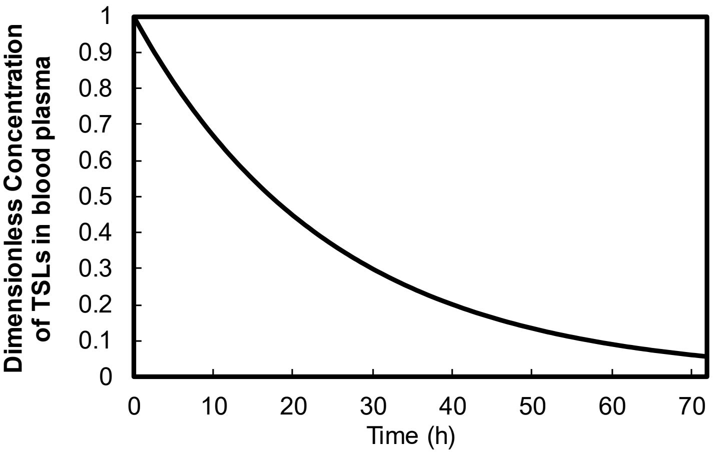

Figure S2. Dimensionless concentration of TSL-Dox in blood circulation system. The concentration gradually decreases from its initial value due to uptake of intravenously injected TSL-Dox by other compartments.

References

1. Salloum, M.; Ma, R.; Zhu, L. Enhancement in treatment planning for magnetic nanoparticle hyperthermia: Optimization of the heat absorption pattern. *Int. J. Hypert.* **2009**, *25*, 309–321, doi:10.1080/02656730902803118.
2. Astefanoaei, I.; Chiriac, H.; Stancu, A. Thermal performance of Fe-Cr-Nb-B systems in magnetic hyperthermia. *J. Appl. Phys.* **2017**, *121*, 104701, doi:10.1063/1.4978313.
3. Ramanujan, S.; Pluen, A.; McKee, T.D.; Brown, E.B.; Boucher, Y.; Jain, R.K. Diffusion and Convection in Collagen Gels: Implications for Transport in the Tumor Interstitium. *Biophys. J.* **2002**, *83*, 1650–1660, doi:10.1016/s0006-3495(02)73933-7.
4. A Peppas, N. Basic Transport Phenomena in Biomedical Engineering: R.L. Fournier, Taylor & Francis, Philadelphia, PA, 1999, 312 pages. *J. Control. Release* **2000**, *67*, 417, doi:10.1016/s0168-3659(00)00218-2.
5. Gao, Y.; Li, M.; Chen, B.; Shen, Z.; Guo, P.; Wientjes, M.G.; Au, J.L.-S. Predictive Models of Diffusive Nanoparticle Transport in 3-Dimensional Tumor Cell Spheroids. *AAPS J.* **2013**, *15*, 816–831, doi:10.1208/s12248-013-9478-2.
6. Stylianopoulos, T.; Jain, R.K. Combining two strategies to improve perfusion and drug delivery in solid tumors. *Proc. Natl. Acad. Sci.* **2013**, *110*, 18632–18637, doi:10.1073/pnas.1318415110.
7. Tang, Y.-D.; Jin, T.; Flesch, R. Effect of mass transfer and diffusion of nanofluid on the thermal ablation of malignant cells during magnetic hyperthermia. *Appl. Math. Model.* **2020**, *83*, 122–135, doi:10.1016/j.apm.2020.02.010.
